# Natural Biomaterials as Instructive Engineered Microenvironments That Direct Cellular Function in Peripheral Nerve Tissue Engineering

**DOI:** 10.3389/fbioe.2021.674473

**Published:** 2021-05-25

**Authors:** Rebecca Powell, Despoina Eleftheriadou, Simon Kellaway, James B. Phillips

**Affiliations:** ^1^UCL Centre for Nerve Engineering, University College London, London, United Kingdom; ^2^Department of Pharmacology, UCL School of Pharmacy, University College London, London, United Kingdom; ^3^Department of Mechanical Engineering, University College London, London, United Kingdom

**Keywords:** peripheral nerve, tissue engineering, biomaterials, microenvironment, regeneration

## Abstract

Nerve tissue function and regeneration depend on precise and well-synchronised spatial and temporal control of biological, physical, and chemotactic cues, which are provided by cellular components and the surrounding extracellular matrix. Therefore, natural biomaterials currently used in peripheral nerve tissue engineering are selected on the basis that they can act as instructive extracellular microenvironments. Despite emerging knowledge regarding cell-matrix interactions, the exact mechanisms through which these biomaterials alter the behaviour of the host and implanted cells, including neurons, Schwann cells and immune cells, remain largely unclear. Here, we review some of the physical processes by which natural biomaterials mimic the function of the extracellular matrix and regulate cellular behaviour. We also highlight some representative cases of controllable cell microenvironments developed by combining cell biology and tissue engineering principles.

## Introduction

Despite significant research and progress in microsurgical techniques over recent decades, peripheral nerve repair remains challenging for clinicians. Recovery from larger gap injuries is especially problematic, with patients commonly living with unsatisfactory restoration of sensory and motor function (Muheremu and Ao, 2015) which can have a substantial impact on quality of life (Wojtkiewicz et al., 2015). Depending on the severity of the injury, a number of interventions may be employed by clinicians to aid the innate regeneration potential of the peripheral nervous system. Where possible, end-to-end suturing of the nerve stumps will be performed following a transection injury. However, when the damage involves a gap which cannot be sutured directly this is not possible, and the gold standard for repair is autologous transplantation of nerve tissue, or autograft. Autografts do come with drawbacks, with patients potentially presenting with morbidity at the donor site, subsequent sensory deficits, neuroma development and having a greater risk of infection. Furthermore, there is a limited amount of available graft material (Lee and Wolfe, 2000). Therefore, a critical need remains for developing effective nerve repair strategies that overcome limitations of autografting.

The design and production of nerve guidance conduits is multidisciplinary and may draw from engineering, biology, and chemistry to provide optimal conditions for the support of axonal regeneration. Nerve repair technology has progressed from the development of simple hollow tubes to more sophisticated engineered tissues, designed to mimic additional features of the nerve autograft. To be successful, engineered tissues must integrate with the host nerve tissue and provide an appropriate environment to support and guide the regeneration of neurons from the proximal to the distal side of the injury site.

There is little consensus in peripheral nerve engineering, despite the wide range of biomaterials available, on which are most suited to supporting cells involved in the repair process such as neurons, Schwann cells, macrophages, and blood vessels. As is the case for many tissue engineering solutions, both natural and synthetic material avenues have been extensively explored with tangible advantages and disadvantages attributed to both options (Zhang et al., 2014; Di Summa et al., 2015; Muheremu and Ao, 2015; Gregory and Phillips, 2021). Synthetic polymers are popular as they can be adapted, through various modifications, to improve cell adhesion and finely tune mechanical properties (Li et al., 2015; Mobasseri et al., 2015; Shahriari et al., 2017). However, natural materials (typically derived from extracellular matrix components) possess innate cell binding motifs, produce harmless degradation products, and effectively invoke natural tissue remodelling and repair pathways (Bonnans et al., 2014; Nicolas et al., 2020). Using extracellular matrix proteins in the form of hydrogels gives the benefit of structural integrity for supporting regenerating axons, whilst maintaining the hydrogel viscoelastic properties and their capability to act as carriers for drugs, growth factors and cells to further improve function (Bhatnagar et al., 2016).

Cell behaviour and nerve extracellular matrix have been characterised extensively. Of particular importance in engineered nerve tissue are interactions between neurons, Schwann cells, macrophages, and endothelial cells with collagen, laminin, and fibronectin due to their presence in native nerve extracellular matrix and the ability of these to be used in hydrogel form. Cells interact with these proteins in an integrin-dependent manner, binding to peptides in fibronectin and laminin such as RGD which is recognised by α5β1 integrins and attaching to collagen via interactions involving α_1_-integrins in particular (Brown and Phillips, 2007; O’Rourke et al., 2015). Fibrin is another protein used in peripheral nerve repair that, although not present in the native extracellular matrix, is deposited after injury. Fibrin forms a nerve bridge joining the two ends of an injured peripheral nerve which acts as early guidance for fibroblasts, endoneurial cells, and repair Schwann cells (Hodde et al., 2016) and therefore has an important role in the peripheral nerve injury response. There have been various studies that have used extracellular matrix protein hydrogels inside outer tubes to influence cell behaviour in animal models, shown in Table 1. Included in the table are those studies which investigated the impact of the biomaterial on the phenotype of cells involved in the repair process–namely Schwann cells, neurons, and macrophages. Both cellular and acellular implanted constructs have been included, in addition to more complex hydrogels such as those releasing growth factors. *In vitro* and *in vivo* experimental outcomes that have shown to have a positive effect on peripheral nerve regeneration are the increased viability, adhesion, proliferation, and elongation of Schwann cells in addition to increased neurite extension from neural cells and the polarisation of macrophages towards an M2, pro-regenerative phenotype (Badylak et al., 2008; Georgiou et al., 2015; Jessen and Mirsky, 2019).

**TABLE 1:** Summary of extracellular matrix hydrogels as nerve guidance conduit luminal fillers in *in vivo* models.

**Conduit**	**Hydrogel material**	**Additional components**	**Implanted cell**	**Species**	**Gap length**	**Cell type of interest**	**Cell behaviour**	**References**
COLLAGEN	Collagen I	None	Differentiated adipose-derived stem cells	Rat	10 mm sciatic	Schwann cells	Collagen-cell combination conduits promoted Schwann cell infiltration, preferentially along the walls of the conduit	Di Summa et al., 2014
COLLAGEN	Collagen	None	Acellular	Dog	35 mm sciatic	Neurons	More nerve fibres and larger myelinated area with NGF versus without, although thinner compared to autograft	Yao et al., 2018
ACRYLONITRILE VINYLCHLORIDE COPOLYMER	Matrigel	None	Primary rat Schwann cells	Rat	8 mm sciatic	Neurons, Schwann cells	Greater number of myelinated axons with Schwann cells, which increased with Schwann cell density, although Matrigel alone was inhibitory. Heterologous induced immune response impeding regeneration	Guenard et al., 1992
CHITOSAN	Collagen	Laminin or fibronectin	Acellular	Rat	15 mm sciatic	Schwann cells	Greatest infiltration of Schwann cells observed in stabilised fibronectin and laminin conduits	Gonzalez-Perez et al., 2017
SMALL INTESTINE SUBMUCOSA	Collagen I	Hyaluronic acid	Acellular	Rat	10 mm sciatic	Schwann cells	Aligned hydrogels resulted in elevated Schwann cell migration and proliferation at 4 weeks	Lacko et al., 2020
CHITOSAN	Collagen I	Laminin or fibronectin	MSCs/Schwann cells	Rat	15 mm sciatic	Neurons, Schwann cells, MSCs	Better functional results in those with implanted Schwann cells in fibronectin-aligned constructs, despite *in vitro* greater proliferation on laminin	Gonzalez-Perez et al., 2018
COLLAGEN	Collagen	Collagen-gag	Acellular	Rat	10 mm sciatic	Neurons	Comparable axon and nerve fibre density between collagen/GAG and autograft, although number of myelinated nerves reduced	Lee et al., 2012
COLLAGEN I	Collagen I	Hyaluronic acid	Neural stem cells	Rabbit	5 mm facial	Neurons, macrophages	No infiltration of macrophages. HA supported developmentally immature neurons and promotes neurite outgrowth	Zhang et al., 2008
POLY-L-LACTIDE-CO-CAPROLACTONE	Collagen I	Hyaluronic acid, NGF	Acellular	Rat	10 mm sciatic	Neurons	Additions of hydrogel to conduits did not improve motor neuron recovery, but sensory neurite outgrowth increased in presence of NGF	Jin et al., 2013
POLY-D,L-LACTATES	Laminin	Collagen IV, heparan sulfate, proteoglycan	Acellular	Mouse	4–5 mm sciatic	Neurons	Laminin had stimulatory effect on axon growth	Madison et al., 1985
COLLAGEN I	Hyaluronic acid	Laminin-simulating peptide, sodium dismutase	Acellular	Rat	15 mm sciatic	Neurons	Myelinated axons, axonal sprouting comparable to autograft	Rochkind and Nevo, 2014
SILICONE	Collagen I	Fibrin gel	Schwann cells	Rat	10 mm sciatic	Neurons, endothelial cells	Blood vessel numbers in GAE-EngNT comparable to autograft, 8-fold more axons in GAE than the empty tube with neural extensions following aligned Schwann cells	Muangsanit et al., 2020
SILICONE	Peripheral-nerve specific extracellular matrix	None	Acellular	Rat	15 mm sciatic	Macrophages, Schwann cells	M1 and M2 macrophages and Schwann cell numbers were increased with PNSECM in addition to the M2:M1 ratio compared to silicone conduit alone	Prest et al., 2017
POLY (L-LACTIC ACID)-CO-POLY (TRIMETHYLENE CARBONATE)	Porcine decellularised nerve matrix	NGF (3.3 μg/ml)	Acellular	Rat	15 mm sciatic		Schwann cell migration increased with porcine decellularised nerve matrix with NGF compared to conduits without NGF	Rao et al., 2021

*GAG, glycosaminoglycans; MSCs, mesenchymal stem cells; NGF, nerve growth factor. The majority of nerve guidance conduits are formed of a stiffer/stronger conduit filled with a soft hydrogel material to give greater structural integrity to the implant, and the hydrogel can be used as an acellular construct or seeded with cells prior to implantation (column 4). *In vivo* models of nerve repair involved the transection of a peripheral nerve, commonly sciatic (column 6), with critical gap lengths (over which no repair is possible without a graft or bridging) of around 15 mm in rats, 30 mm in rabbits and dogs and 40 mm in pigs and humans. The studies included were those that implanted conduits *in vivo* with an extracellular matrix luminal filler surrounded by an outer tube, and measured outcomes related to the behaviour of key cells involved in the repair process–Schwann cells, macrophages and neurons (columns 7 and 8).*

**FIGURE 1:**
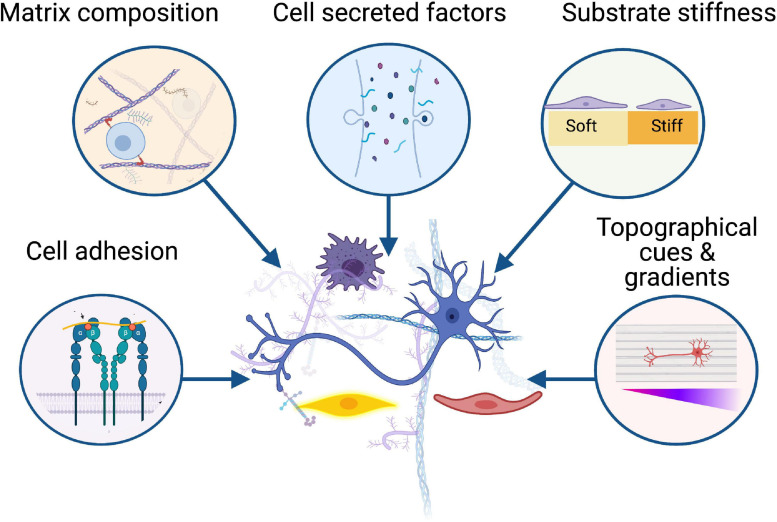
Biomaterials influence the behaviour of key cellular players in peripheral nerve regeneration including neurons, Schwann cells, immune cells, and vasculature. Successful regeneration is dependent on the interactions between these cells and the surrounding microenvironment, from cell adhesion to substrate stiffness, growth factors, and topographical gradients. Created with BioRender.com.

**FIGURE 2:**
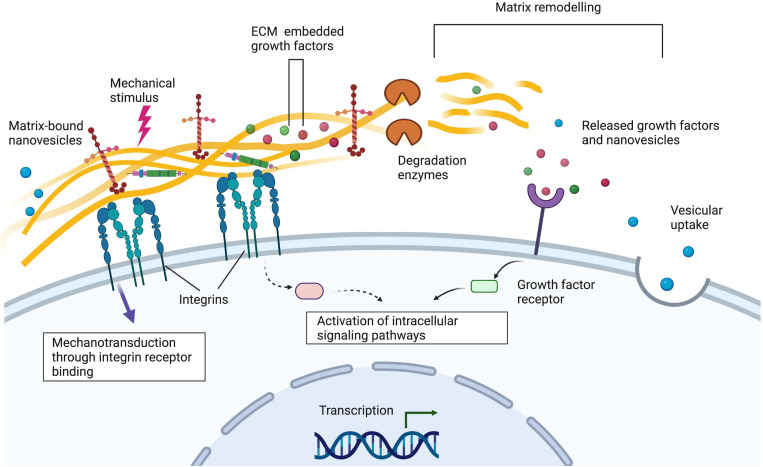
Integrins on the cell surface bind to extracellular matrix proteins such as laminin and collagen. Alterations to the extracellular matrix via matrix remodelling, the addition of matrix-bound nanovesicles and growth factors and mechanical stimuli can alter cellular behaviour. Understanding these interactions is important when designing nerve guidance constructs for peripheral nerve tissue engineering applications. Created with Biorender.com.

In this review, we have identified common extracellular matrix proteins used in hydrogels for peripheral nerve repair applications and the way in which these can mimic the function of native extracellular matrix and regulate cellular behaviour (Figures 1, 2). We highlight some representative examples of controllable cell microenvironments developed by combining cell biology and tissue engineering principles which will be useful for future development of cellular nerve guidance conduits. Understanding how these different cell types interact with extracellular matrix protein hydrogels will allow us to choose the most appropriate materials for nerve guidance conduits and hopefully improve outcomes for translational therapies.

## Cellular Behaviour Supporting Peripheral Nerve Regeneration

Schwann cells provide neurotropic support and are involved in the inherent regeneration response. They transform after denervation into repair Schwann cells which proliferate and elongate to form Bands of Büngner which support and guide the regenerating axons (Deumens et al., 2010; Jessen et al., 2015; Jessen and Mirsky, 2016). Without this Schwann cell guidance, undirected growth of axons can occur resulting in unsuccessful repair alongside muscle atrophy at the distal target and neuroma formation at the proximal stump (Deumens et al., 2010; Gomez-Sanchez et al., 2015; Jessen et al., 2015; Jessen and Arthur-Farraj, 2019). Accelerating organised Schwann cell migration may be critical in improving axonal regeneration throughout the conduit (Chen et al., 2019). Ensuring viability and the spatial organisation of supporting cells is important to the success of the nerve guidance conduit in peripheral nerve regeneration (Smith and Stevenson, 1988). Repair Schwann cells are also an integral part of many other intercellular interactions (Pan et al., 2020), for example the recruitment of macrophages, which aid in the formation of a pro-regenerative environment in addition to releasing VEGF to encourage the growth of blood vessels (Lindborg et al., 2017; Roberts et al., 2017; Zigmond and Echevarria, 2019). Blood vessels in turn help guide the Schwann cells across the nerve bridge and supply oxygen to the injury area (Cattin et al., 2015). If oxygen deprivation is prolonged, the repair Schwann cells, neurons and other cell types in the regenerative region will die and impair axon regeneration (Krock et al., 2011). Schwann cells also remyelinate the regenerated axons, allowing complete reinnervation (Stratton et al., 2018). Supporting Schwann cell survival and proliferation alongside blood vessel formation is therefore likely to be essential to the overall success of nerve guidance constructs.

Another aspect to consider is the role of immune cells such as macrophages in the peripheral nerve regeneration process. Inflammatory events following peripheral nerve injury are complex (Dubový et al., 2013). Initially it is crucial macrophages phagocytose cellular and extracellular debris, however, like many other tissues, regeneration of the peripheral nerve is impaired until inflammation is resolved with the activation of the anti-inflammatory immune response (Ydens et al., 2012). Over-activation of the inflammatory response will result in the formation of fibrotic tissue at the injury site which impairs regeneration as regenerating axons and supporting Schwann cells cannot penetrate fibrotic scars (Wang et al., 2019). The differing macrophages can be characterised broadly as M1 (inflammatory) or M2 (anti-inflammatory). The employment of materials that promote the polarisation of macrophages towards the M2 phenotype is often desired but must be carefully timed to allow initial clearance of debris.

## The Addition of Laminin or Fibronectin and Certain Peptides Can Improve Functional Outcomes

Laminin, fibronectin and other components have been added to collagen hydrogels to try and improve cell proliferation and the regenerative potential of the biomaterials. Schwann cells both deposit and interact with laminin (Chernousov and Carey, 2000). Schwann cell migration across the nerve bridge is also aided by the presence of laminin (Mccarthy et al., 1983; Motta et al., 2019), as well as it being a key factor affecting the ability of Schwann cells to sense and respond to mechanical properties of matrices (Urbanski et al., 2016). Mice with Schwann cells expressing disrupted laminin γ1 display impaired peripheral nerve myelination and regeneration (Chen and Strickland, 2003), suggesting the presence of laminin in the nerve bridge is important for successful regeneration. Suri and Schmidt (2010) added 100 μg/ml laminin to collagen and hyaluronic acid hydrogels and found the release of neurotrophic factors, proliferation rate and metabolic activity was increased from the encapsulated primary rat Schwann cells. This was compared with both cell-laden collagen and hyaluronic acid hydrogels containing lower concentrations of laminin, and Schwann cells encapsulated in collagen-only hydrogels (Suri and Schmidt, 2010). Although this study shows the presence of laminin has a positive effect on Schwann cell behaviour, this nerve guidance construct was not implanted and it is therefore unclear if and how this effect will translate *in vivo*.

The use of extracellular matrix peptides may be relevant for translational therapies as synthesising peptides is more reproducible than isolating natural extracellular matrix molecules. In addition, specific cell functions may be targeted by carefully choosing the peptides (Boontheekul and Mooney, 2003). In fact, the active peptides of laminin–those important for Schwann cell adhesion–have also been isolated and combined with hydrogels to try to improve the behaviour of neurons and other cell types (Hiraoka et al., 2009; Hosseinkhani et al., 2013; Rochkind and Nevo, 2014; Motta et al., 2019). Motta et al. (2019) compared a number of these and assessed the proliferation and adhesion behaviour of Schwann cells alongside that of fibroblasts. Hosseinkhani et al. (2013) also looked at the laminin peptides IKVAV and RGD on dorsal root ganglia (DRG) proliferation and differentiation. They found collagen matrices modified with the peptides RGD, IKVAV, and RGD-IKVAV increased attachment, proliferation and increased potential to differentiate in media with acidic fibroblast growth factor (aFGF). Including IKVAV had the most significant results in all outcomes compared to RGD alone, as well as collagen modified with VVIAK and those on 2D culture. Taken together, these studies indicate the laminin peptide IKVAV has positive effects *in vitro* on both Schwann cells and neurons. Immune cells are also able to bind to and be affected by cryptic peptides in collagen such as discoidin domain receptors (DDR) 1 and 2, leukocyte-associated immunoglobulin-like receptor 1 (LAIR-1) as well as RGD, suppressing immune cell activity. Fibrin also contains P1, P2, and RGD peptides which immune cells can interact with via αMβ2 integrins (Rowley et al., 2019), with fibrin inducing both inflammatory and anti-inflammatory responses.

Using materials which encourage longer processes to form in implantedcells can also aid the growth of axons over a longer distance. Gonzalez-Perez et al. (2017) developed aligned constructs formed of laminin or fibronectin blended with collagen 1 hydrogel and contained these in a chitosan conduit. Later, this construct was seeded with Schwann cells or mesenchymal stem cells and implanted into a 15 mm sciatic nerve gap in rats (Gonzalez-Perez et al., 2018). Improved functional outcomes, such as electrophysiology, were seen with fibronectin and laminin as well as an increased number of axons and myelinated fibres when compared to collagen alone (Gonzalez-Perez et al., 2017). Schwann cells also migrated to a slightly greater degree in these blends although this was not significant compared to collagen alone (Gonzalez-Perez et al., 2017). Fibronectin and laminin seem to induce generally similar outcomes in cell behaviour, although the *in vivo* cellular constructs with fibronectin and rat Schwann cells showed a greater increase in motor neuron reinnervation compared to laminin or those containing mesenchymal stem cells. In addition, at the distal nerve end, the fibronectin and Schwann cell constructs had a significant increase in myelinated fibres (Gonzalez-Perez et al., 2018). This aligns with *in vitro* data that showed Schwann cells elongated to a greater extent on fibronectin compared to laminin (Gonzalez-Perez et al., 2017, 2018), indicating that Schwann cell elongation is associated with positive effects on neuron growth. Stem cells have been widely used either directly in peripheral nerve guidance constructs or differentiated into Schwann cell-like cells with promising results (Ziegler et al., 2011; Martens et al., 2014; Georgiou et al., 2015; Sakaue and Sieber-Blum, 2015; Jung et al., 2016; Cai et al., 2017; Huang et al., 2017, 2020; Kim et al., 2017; Sanen et al., 2017; Jones et al., 2018; Kubiak et al., 2020). However, it is not known in these cases if these differentiated cells will behave comparably in response to different biomaterials to the Schwann cells found in a native injury environment.

Another more complex hydrogel addition to nerve guidance conduits is Matrigel, a basement membrane hydrogel containing laminin, collagen IV, heparan sulphate proteoglycans and growth factors, which is commonly used as a cell carrier and coating for neural stem cells (Rodriguez et al., 2000; Kim et al., 2007; Koutsopoulos and Zhang, 2013; Li et al., 2018; Liu et al., 2020). Including Matrigel in collagen conduits has been shown to improve regeneration compared with collagen alone, which was not improved with the addition of Schwann cells (Udina et al., 2004), as well as encouraging Schwann cell infiltration into the proximal and distal device of a hollow conduit, resulting in improved axonal regeneration in short gap repair models (Madison et al., 1985; Labrador et al., 1998). Although Matrigel supports neural cells and Schwann cell migration *in vitro* it is unclear if this translates well to *in vivo* peripheral nerve regeneration as in other studies the inclusion of Matrigel decreased axon number compared to the empty tube control or autograft (Guenard et al., 1992). The source and variability of this material mean it would also not be appropriate for clinical therapy.

As previously discussed, the M2 macrophage response is important to successful regeneration. Macrophages also express integrins which allow them to bind to the RGD peptides found in laminin and fibronectin. Binding of integrins activates macrophages and induces the release of pro-inflammatory cytokines such as Tumour Necrosis Factor-alpha (TNF-α) and Interleukin 6 (IL-6). In addition, they can respond to biomaterials too large to phagocytose by binding together to form foreign body giant cells (Anderson et al., 2008; Sheikh et al., 2015). Blocking the RGD-binding integrins on macrophages resulted in a thinner foreign body capsule, as well as reduced release of inflammatory cytokines in response to lipopolysaccharides (Zaveri et al., 2014). This study suggests the binding of macrophages to biomaterials expressing RGD peptides via integrins may result in the release of inflammatory cytokines, which could be detrimental to regeneration. This is in contrast to those studies suggesting the presence of RGD peptides are beneficial due to the interaction between neurons and Schwann cells.

In addition to protein hydrogels increasing neurotrophic factor release, metabolic activity and adherence of Schwann cells, the addition of guiding fibres from fibrillar collagen or synthetic compounds can aid cell elongation reminiscent of the repair Schwann cell phenotype. Repair Schwann cells interact with fibrin deposited in the nerve bridge after injury, and when Schwann cell proliferation was assessed in response to a two-dimensional fibrin substrate versus a fibrin hydrogel, there was found to be increased Schwann cell proliferation in two-dimensional cultures. However, the Schwann cells in fibrin hydrogels formed more complex branching which may indicate a greater potential to form Bands of Büngner-like structures despite the lower proliferation rates (Hodde et al., 2016). With polycaprolactone aligned fibres included in the hydrogel, the Schwann cells also extended long processes. Schuh et al. found a collagen-fibrin blend containing 10% fibrin and 90% collagen type I promoted Schwann cell viability compared with collagen alone. *In vitro*, there was also shown to be increased neurite viability, alignment and outgrowth of NG108 cells (a neuroblastoma cell line) on the collagen-fibrin gels. Both of these studies translated to *in vivo* experiments showing increased axonal regeneration (Hodde et al., 2016; Schuh et al., 2018), suggesting that fibrin increases the viability of Schwann cells, but additional guiding fibres such as the polycaprolactone and fibrillar collagen are required to allow longer processes to form and contribute to regenerating axonal guidance.

Alternatively, biologically complex materials may be produced via the decellularisation of animal tissues and their subsequent processing to form extracellular matrix hydrogels. When implanted *in vivo*, these materials provide site appropriate extracellular matrix components and modulate cellular behaviour, such as macrophage phenotype, to promote positive tissue remodelling (Crapo et al., 2011; Medberry et al., 2013; Saldin et al., 2017; Spang and Christman, 2018). Tissue specific effects of hydrogels derived from decellularised extracellular matrix have been observed in neural tissue repair; peripheral nerve extracellular matrix derived hydrogels were biochemically distinct from spinal cord extracellular matrix derived hydrogels and thus had specific effects on the transcriptome of neural stem cells (Xu et al., 2021). Prest et al. (2017) employed a canine peripheral nerve derived extracellular matrix hydrogel within a silicone conduit that improved Schwann cell infiltration and increased the M2:M1 macrophage population ratio when compared to an empty conduit in a critical sized defect. Moreover, decellularised extracellular matrix hydrogels may act as a growth factor reservoir, improving their efficacy *in vivo* as controlled delivery systems (Briquez et al., 2015; Martino et al., 2015a, b). For example, Rao et al. (2021) reported elevated Schwann cell migration at the proximal and distal stumps in conduits filled with porcine derived peripheral nerve extracellular matrix hydrogels when they were bound with NGF. Furthermore, synergistic effects were observed in the proliferation and migration of Schwann cells using peripheral nerve derived extracellular matrix hydrogels as a system for the codelivery of VEGF and NGF resulting in improved functional recovery (Li et al., 2020) after sciatic nerve crush.

## Effect of Stiffness on Macrophages and Schwann Cells

Recent studies examined how the local microenvironment influences human macrophage M1/M2 polarisation, with polarisation toward the M2 phenotype being induced by increasing the stiffness of the substrate (Friedemann et al., 2017). Friedemann et al. embedded human macrophages in 3D porous type I collagen hydrogels and found that the higher the rigidity of the matrix (27–100 Pa), the higher the secretion of Interleukin-10 (IL−10) and the lower the secretion of TNF-α and Interleukin-12 (IL−12). These changes in the cytokine expression profiles are indicative of a more pro-regenerative macrophage phenotype. However, a subsequent study using 3D collagen gels with elastic moduli equivalent to 0.5–1.5 kPa did not observe any consistent differences in the macrophage response to the stiffness of the scaffolds. Instead, the authors demonstrated a clear correlation between macrophage polarisation and the cross-linking method employed to alter the mechanical properties of the scaffold (Sridharan et al., 2019). 3D matrix architecture, rather than stiffness, was also found to be the major factor directing macrophage migration through collagen hydrogels (Van Goethem et al., 2010). For instance, the fibril density of collagen hydrogels has been shown to control macrophage activation and infiltration (Sapudom et al., 2020), creating a complex picture of macrophage behaviour and hydrogel mechanical properties.

Several studies have shown that Schwann cells can develop normally on both stiff and soft hydrogels but activate different intracellular pathways in response to different substrate stiffness. Urbanski et al. (2016) found no significant differences in Schwann cell attachment or viability between soft (1.5 kPa) and rigid matrices (30 kPa), while a slight increase in proliferation was observed on the rigid substrates. This limited response may be expected as under physiological conditions, Schwann cells have to adapt to the wide range of elastic moduli that may occur in the native microenvironment. Variations in extracellular matrix elasticity were also found to induce some changes in Schwann cell morphology, albeit less significantly compared to other glial cell types. When cultured on soft substrates enriched with collagen and laminin, Schwann cells acquired a more elongated phenotype and exhibited more actin-rich processes along the membrane. On rigid matrices, Schwann cells adopted a more flat and polygonal shape. Adjusting the elasticity of hydrogels to promote elongated phenotypes that could potentially form structures mimicking Bands of Büngner may improve the support of axon regeneration by these implanted cellular hydrogels.

Mechanical stimuli have also been found to affect the expression of downstream Hippo pathway co-activators Yap and Taz in Schwann cells, which in turn induce the expression of basal lamina receptor genes. These co-activators are vital for radial sorting of axons and subsequent myelination (Poitelon et al., 2016)–important at the later stages of injury when remyelination of the regenerated axon occurs (Taveggia et al., 2010; Stratton et al., 2018). Interestingly, similar to the limited responsiveness of Schwann cells to substrate stiffness, there seems to be a threshold after which Schwann cells appear to be unaffected–they showed insignificant morphological changes and no variations in the expression of YAP/TAZ mechanotransducers from 40 kPa to 4 MPa (Poitelon et al., 2016). The presence of laminin again can greatly change Schwann cell response to mechanical stimuli as it acts as a co-factor in activating YAP/TAZ mechanotransducers (Poitelon et al., 2016).

Neurite extension is affected by both interfibre spacing and mechanical stiffness within collagen hydrogels (Willits and Skornia, 2004). DRG neurite outgrowth on three-dimensional (3D) collagen hydrogels was shown to be biphasic and increased on softer to intermediate substrates (<10 Pa). The slower neurite extension rates observed on more stiff collagen gels could be linked to the denser fibre network (Willits and Skornia, 2004). DRG co-cultures are commonly used to model the growth of neurons on top of cellular and acellular constructs, as an indicator of the regenerative potential (Bozkurt et al., 2007; Kingham et al., 2011; Stettner et al., 2013). DRG explants are also useful as a model of myelination as they contain a mixed population of neurons and glial cells (Hanani, 2005; Emery and Ernfors, 2018), with the neurons in DRGs known to regenerate their axons actively after injury (Khoshakhlagh et al., 2018). Matrix elasticity also regulated neurite outgrowth in fibrin gels (Man et al., 2011). More recently, Nichol et al. demonstrated that human motor neurons differentiated from induced pluripotent stem cells generate longer neurites and extend faster within more rigid three-dimensional collagen I hydrogels (∼5 kPa). The authors suggested that the strong adhesion of leading-edge protrusions was responsible for accelerated neurite extension, and correlated with increased activation of ras homolog gene family member A (RHOA) expression (Nichol et al., 2019). RHOA signalling could thus play an important role in controlling mechanosensitive neuronal responses in elastic environments. The results of the previous studies indicate that the regulation of human neuritogenesis by 3D environmental elasticity may differ depending on neuronal subtype. It is nevertheless important to highlight that the methods of characterising the mechanical properties of hydrogels in these studies are very diverse, which makes direct comparisons of the results more challenging.

## Stiffness Gradients and Topographical Features Direct Neurite Outgrowth and Schwann Cell Elongation

The presence of stiffness gradients can also affect neurite extension, cell migration, and axonal branching. Guided cell migration is a key element in neural tissue engineering, where recent efforts have focused on strategies that guide neurons and support cells across gaps resulting from peripheral nerve injury (Wrobel and Sundararaghavan, 2013). Using a microfluidic system, Sundararaghavan et al. studied the durotactic response of neurites from chick DRG explants to 3D collagen gels with tailored mechanical gradients of varied stiffness. Results indicate that the presence of gradients can enhance and orientate growth, with the length of neurites growing down the steeper gradient of 377 ± 25 − 57 ± 2.8 Pa being increased, without altering cell adhesion (Sundararaghavan et al., 2009). In collagen hydrogels containing substrate gradients, Kayal et al. (2020) found the extension of neurites from NG108-15 neural cells was dependent on both the gradient and absolute stiffness of the hydrogel. This study highlights again the importance of considering both cues–gradient and stiffness–in controlling orientation of neurites.

Experimental observations have also demonstrated that topographical features such as matrix alignment could potentially encourage nerve regeneration by manipulating and directing neurite outgrowth (Ceballos et al., 1999). For instance, the uniaxial orientation of collagen fibres in magnetically aligned collagen gel rods has been shown to increase the invasion of neurites and their preferential elongation *in vitro*, a response likely caused by contact guidance of growth cones at the neurite tips (Dubey et al., 1999). Similarly, chick DRG explants cultured on aligned freeze -dried collagen scaffolds exhibited preferential outgrowth along the length of the aligned fibres (Lowe et al., 2016). It should be noted when comparing studies using DRGs, that overall embryonic DRGs have a higher regenerative potential compared to adult ones, with embryonic chick DRGs undergoing earlier pseudo-unipolarisation compared to their rat counterparts (Matsuda et al., 1996).

Finally, Schwann cell migration can be guided by different durotactic or topological cues, a response that can be vital for nerve regeneration (Bruder et al., 2007; Evans et al., 2018). Long-term culture (after 21 days) of DRG explants on highly oriented 3D collagen scaffolds showed that migrating Schwann cells aligned within the guidance channels and self-assembled in columns that resembled Bands of Büngner (Bozkurt et al., 2009).

## Adding Neurotrophic Factors Has Shown to Be Beneficial Within Certain Concentration Ranges

Neurotrophic factors are released by repair Schwann cells and contribute to the creation of a pro-regenerative environment in the nerve bridge. The inclusion of these and other growth factors in hydrogels may boost neurotrophic support. Overexpression of growth factors such as glial cell line-derived neurotrophic factor (GDNF) can, however, result in axonal entrapment (Eggers et al., 2019), so any inclusion or release of neurotrophic factors must be carefully controlled in peripheral nerve repair. Allodi et al. (2011) found motor and sensory neurons increase their outgrowth if brain-derived neurotrophic factor (BDNF) or nerve growth factor (NGF) was added to collagen type I hydrogels, respectively. Although Schwann cells were included in experiments, this was only to assess if they could associate with neurons rather than directly measuring the effect of the neurotrophic factors on them (Allodi et al., 2011). NGF has also been incorporated in a fibrin and heparin hydrogel and implanted into rat sciatic nerve gaps (Lee et al., 2003). The NGF was released in a controlled manner and the number of nerve fibres increased corresponding to the NGF concentration up to 2 ng/ml, with no significant differences found between 20 and 50 ng/ml. The addition of NGF to hydrogels may especially target sensory rather than motor neuron regeneration to improve functional outcomes. *In vitro*, DRG outgrowth was impacted in a dose-dependent manner by NGF carried in collagen and hyaluronan hydrogels on a poly-L-caprolactone (PLCL) scaffold developed by Jin et al. (2013) Although this did not translate to significant effects on motor recovery *in vivo*, this study did find NGF improved sensory recovery *in vivo*. The addition of NGF has also been shown to improve regeneration over longer gaps. Yao et al. (2018) contained NGF in a longitudinally orientated collagen conduit (LOCC) which was used *in vivo* to repair a 35 mm nerve gap in dogs. Here, gastrocnemius muscle mass was significantly higher in the LOCC/NGF group than the LOCC alone group.

The effect of including growth factors such as basic fibroblast growth factor (FGF-2) in hydrogels has a positive effect on neuron growth (Sakiyama-Elbert and Hubbell, 2000; Al-Zer et al., 2015; Fujimaki et al., 2017). Indeed, although Dietzmeyer et al. (2020) saw an impairment of regeneration when Schwann cells modified to overexpress FGF-2 were present in their hyaluronic acid and laminin conduit versus the acellular conduits, it was found the inclusion of the Schwann cells had instead induced a downregulation of neurotrophic factor expression compared to that of the *in vitro* 2D culture. Sakiyama-Elbert and Hubbell (2000) additionally modelled the release of FGF-2 from fibrin hydrogels and found its inclusion would improve neurite extension as long as the immobilised heparin it was bound to was in 500-molar excess relative to the FGF-2. FGF-2 may positively impact peripheral nerve regeneration but, similar to neurotrophic factors, only if release is controlled.

## Conclusion

Complex structural support in nerve guidance conduits has been extensively studied with the development of porous materials, and those with internal supporting and guiding structures (Konofaos and Ver Halen, 2013; Bozkurt et al., 2016; Meyer et al., 2016; Belanger et al., 2018). This review is, to our knowledge, the first to focus on how common nerve guidance constructs made with extracellular protein hydrogels alter the phenotype of cells involved in the nerve injury process (Figure 2). Unfortunately, there is very little in common in terms of outcome measures between studies, so it is difficult to compare results directly and any conclusions should be treated with a suitable degree of caution. Still, there is some consensus that the addition of laminin or fibronectin improves functional outcomes by increasing Schwann cell survival and release of neurotrophic factors, as well as making the cells more able to sense and respond to the surrounding mechanical properties (Suri and Schmidt, 2010; Hosseinkhani et al., 2013; Urbanski et al., 2016). There is also an indication that relatively soft gels promote a desirable macrophage response (Van Goethem et al., 2010; Sridharan et al., 2015, 2019), with Schwann cells being less sensitive to mechanical changes (Urbanski et al., 2016). In terms of neurons, it may be beneficial to create gels with a gradient of stiffness or aligned fibres to improve guidance and increase neurite extension (Ceballos et al., 1999; Dubey et al., 1999; Sundararaghavan et al., 2009). Adding neurotrophic factors has also shown to be beneficial although concentration plays a critical role in this and, like with mechanical cues, the response is dependent on neuronal subtype (Allodi et al., 2011; Jin et al., 2013; Yao et al., 2018; Nichol et al., 2019).

Understanding the behaviour of cells in response to alteredbiomaterials is an essential consideration when combining cells and materials in tissue engineering efforts, and more research should be undertaken in this area. We acknowledge the focus of this review on extracellular matrix protein hydrogels–chosen due to the role of these in the innate peripheral regeneration response and common use of these materials in peripheral nerve guidance constructs–does therefore not include a host of information on cellular behaviour in response to other hydrogels such as alginate, as well as synthetic constructs. Naturally derived extracellular matrix, such as those from bone, small intestine and bladder, of course contain all the proteins mentioned here and therefore the information in this review may be found to be applicable.

## Author Contributions

All authors contributed equally to preparing, advising, and approving the manuscript.

## Conflict of Interest

The reviewer KH-T declared a past collaboration with several of the authors (RP and JP) to the handling editor. The remaining authors declare that the research was conducted in the absence of any commercial or financial relationships that could be construed as a potential conflict of interest.
